# An Enhanced Jaya Algorithm with Mutation and Diversity-Preserving Strategies for Hyperspectral Band Selection

**DOI:** 10.12688/f1000research.167794.2

**Published:** 2026-01-24

**Authors:** Suchismita Behera, Partha Pratim Sarangi, Bhabani Shankar Prasad Mishra

**Affiliations:** 1 School of Computer Engineering, Kalinga Institute of Industrial Technology, Bhubaneswar, Odisha, 751024, India; 2 School of Computer Engineering, Kalinga Institute of Industrial Technology, Bhubaneswar, Odisha, 751024, India; 3 School of Computer Engineering, Kalinga Institute of Industrial Technology, Bhubaneswar, Odisha, 751024, India

**Keywords:** Band selection, binary Jaya algorithm, mutation operator, hyperspectral image classification.

## Abstract

Hyperspectral band selection has become a key focus in hyperspectral image processing as it reduces the spectral redundancy and computational overhead, thereby improving classification performance. However, optimal band selection remains challenging due to its combinatorial nature. Although numerous metaheuristic algorithms have been introduced in recent years to address this problem, achieving an effective balance between exploration and exploitation continues to pose a major challenge. This paper proposes a novel approach that combines a parameter-free binary Jaya algorithm with a mutation operator to enhance exploration and maintain solution diversity within the search space. We employ Opposition-based Learning (OBL) for population initialization and Quasi-Reflection reinitialization strategy to add diversity whenever fitness stagnation occurs. To simultaneously improve classification performance and band reduction we adopt weighted sum multi-objective fitness function that minimizes redundancy and enhances model generalization. Our proposed method is evaluated using three benchmark datasets, namely Indian Pines, Pavia University, and Salinas. Experimental results demonstrate that the pro-posed method outperforms recent metaheuristic-based band selection techniques. Its superior performance makes it well suited for various HSI applications.

## Introduction

Hyperspectral imaging (HSI) collects detailed spectral data across hundreds of continuous, narrow wavelength bands, providing significant potential for applications like agriculture, remote sensing, and environmental monitoring. ^1^ Despite their rich spectral information, hyperspectral images come with high dimensionality, which introduces several challenges—ranging from increased computational load to the inclusion of redundant, irrelevant data and unwanted noise. Band selection ^2^ is a widely utilized dimensionality reduction technique ^3^ which tackles these challenges by choosing a subset of the most informative spectral bands, thereby enhancing computational efficiency and boosting classification accuracy. ^4^

In the past few decades, band selection in hyperspectral imaging has gained significant attention due to the need to reduce the high-dimensional nature of the data while preserving critical information. Conventional techniques ^5^ often find it difficult to navigate the vast and complex search space, where the most informative bands are dispersed irregularly and are not grouped in a continuous manner. Machine learning approaches: such as Random Forests (RF), ^6^ Support Vector Machines (SVM), ^7^ and unsupervised techniques like Principal Component Analysis (PCA), ^8^ utilize underlying data patterns to carry out both feature selection and classification concurrently. These data-driven techniques are highly adaptable but often necessitate preprocessing steps, such as band selection, to handle high-dimensional spaces effectively. To address this, various metaheuristic algorithms ^9– 11^ have been proposed as effective alternatives for identifying the most informative bands. These algorithms, provide robust solutions by navigating the complex optimization landscape, ensuring efficient and accurate band selection for a wide range of hyperspectral applications. To achieve optimal band selection, a combination of metaheuristic algorithms ^12– 16^ and machine learning methods ^17^ has been extensively investigated. Metaheuristic algorithms, ^18^ such as Genetic Algorithms (GA) ^9, 19^ models’ genetic inheritance, passing advantageous traits to subsequent generations of solutions, Particle Swarm Opti-mization (PSO) ^10, 20^ operates on swarm intelligence principles, replicating the movements of bird flocks or fish schools, and Grey Wolf Optimization (GWO) ^11, 21^ takes inspiration from the natural leadership structure and cooperative hunting strategies of grey wolves. Because it can effectively search through wide and complex solution spaces, it proves to be especially useful for finding the most suitable set of spectral bands. These methods excel at balancing exploration (identifying novel solutions) and exploitation (refining known effective solutions) ^22^ through iterative processes, ensuring robustness even with high-dimensional data and limited prior knowledge. Hybrid approaches ^23^ combining metaheuristic algorithms with machine learning methods have recently emerged, offering improved convergence rates and solution quality for complex hyperspectral imaging tasks.

The existing literature reveals several research gaps, including how initial solutions are selected, how thoroughly the solution space is explored, and how well diversity within the population is preserved. Therefore, in this study, we introduce an improved version of the Jaya algorithm aimed at selecting the most effective subset of bands for hyper-spectral images, with the goal of avoiding local optima and significantly boosting classification performance. A balance between exploration and exploitation is stroked by our approach to effectively reach the global optimum within a high-dimensional search space, the approach demonstrates outstanding performance and has outperformed several existing methods in recent studies. The Jaya algorithm ^24^ is well-known for its simplicity and for being a parameter-free optimization algorithm. We employed the Jaya algorithm in this study to enhance exploitation of the search space and eliminate the need for hyperparameter tuning. The proposed method, which integrates strategies aimed at enhancing population diversity and exploration, contributes significantly to overall effectiveness.

The main highlights and contributions of this work can be outlined as follows:

• We propose a wrapper-based band selection (BS) method, EJaya, a binary Jaya algorithm enhanced with a mutation operator to increase population diversity while avoiding premature convergence. This leads to the selection of more informative and compact spectral band subsets.
• The initial population is generated using Opposition-Based Learning (OBL) ^25^ to ensure better diversity and proximity to optimal solutions. To prevent stagnation, a Quasi-reflection reinitialization (QRI) ^26^ mechanism periodically generates diverse candidates that enhances global search in the solution space.
• We introduce a multi-objective fitness function that combines classification accuracy and spectral class separability (measured using the Jeffries–Matusita distance). This ensures that the selected bands are not only optimal for classification but also maximally discriminative. Additionally, elite preservation and an early stopping criterion are integrated to retain optimal solutions and reduce unnecessary computation.
• We compare the proposed enhanced Jaya-based approach with several widely used metaheuristic-driven band selection techniques evaluated using three standard hyperspectral datasets: Indian Pines, Pavia University, and Salinas. The results demonstrate superior classification accuracy with fewer selected bands.

The rest of this paper is structured as follows: related works has been explained in Section 2, a comprehensive ex-planation of the methodology used in this study is provided in Section 3, while a detailed analysis of the experiments performed is explained in Section 4. Finally, Section 5 presents our conclusions along with possible directions for future research.

## Related work

The evolution of metaheuristic algorithms for hyperspectral band selection ^2, 27^ provides a rich context for understanding the advances in this challenging field. Hyperspectral images (HSIs), with their hundreds of highly correlated spectral bands, impose significant computational and analytical burdens that necessitate effective dimensionality reduction techniques. Metaheuristic algorithms, with their flexible and robust optimization capabilities, have been extensively applied to select optimal bands that maximize classification accuracy while reducing redundancy and noise. We subsequently offer a comprehensive in-depth chronological overview of key metaheuristic algorithms employed for band selection, focusing on their methodologies, limitations, and how the methods have influenced subsequent research developments.

• Genetic Algorithm (GA) are among the earliest and most widely studied evolutionary techniques for hyperspectral band selection. ^28, 29^ They simulate natural evolution using selection, crossover, and mutation to evolve band subsets, typically represented as binary strings or continuous vectors. GAs often relies on wrapper-based fitness evaluation using classifiers like SVM to guide the search toward informative bands. While effective, GAs can be computationally intensive for high-dimensional data and require careful tuning of parameters to maintain diversity and avoid premature convergence. Despite these challenges, GAs have shown strong performance in agricultural and disease detection tasks and have inspired continuous and hybrid variants, establishing them as a benchmark in hyperspectral analysis.
• Particle Swarm Optimization (PSO) introduces a swarm intelligence-based strategy draws inspiration from the coordinated movements seen in flocks of birds and schools of fish. In hyperspectral band selection, each particle represents a potential subset of bands and adjusts its position by learning from its own best experience and the best performance in the group, aiming to enhance classification accuracy, typically using SVM-based fitness evaluations. ^20^ PSO is favored for its simplicity and faster convergence compared to GA. ^30^ Nevertheless, it faces notable limitations, including a tendency to converge prematurely to local optima, limited robustness when handling high-dimensional or noisy datasets, and significant sensitivity to parameters like inertia weight and acceleration factors. These issues are particularly problematic when dealing with nonlinearly separable or highly correlated spectral features. While hybrid and adaptive variants of PSO have been proposed to mitigate these weaknesses, standard PSO often trades off solution quality for speed, making it less stable than GA in complex HSI scenarios.
• Grey Wolf Optimizer (GWO) introduces a swarm intelligence approach structured around a hierarchical model, inspired by the cooperative hunting patterns of grey wolves. The search for optimal band subsets is directed by the leading wolves—alpha, beta, and delta as described in. ^31, 32^ It balances exploration and exploitation through encircling and attacking mechanisms, making it well-suited for high-dimensional hyperspectral band selection. GWO has shown competitive performance on datasets like Indian Pines and Pavia University, often producing compact subsets without sacrificing classification accuracy. However, it relies heavily on parameter tuning to maintain population diversity and can suffer from local optima in later iterations. Additionally, its computational cost increases with feature space size. These limitations have prompted hybrid versions with improved initialization and mutation strategies, highlighting GWO’s adaptability but also its sensitivity to configuration in complex HSI scenarios.
• Ant Colony Algorithm (ACA) model band selection as a path-finding problem, where artificial ants construct band subsets guided by pheromone trails and heuristic cues such as mutual information or redundancy scores. ^33^ Pheromone updates balance exploration and exploitation, aiming to avoid stagnation and guide ants to-ward optimal subsets. ^34^ While ACA promotes solution diversity and heuristic-driven search, it suffers from slow convergence and risks premature convergence due to pheromone saturation. Its performance is highly sensitive to parameter tuning—especially evaporation rate and heuristic weight—and pheromone update steps can be computationally expensive for large hyperspectral datasets. Improved variants have sought to enhance convergence speed and diversity control, ^35^ but ACA remains less efficient than other swarm-based methods, despite its biological inspiration and strong heuristic incorporation.
• Moth-Flame Optimization (MFO) simulates the transverse orientation behavior of moths, using spiral trajectories to explore the solution space around elite solutions (flames). ^36, 37^ Its adaptive flame-reduction mechanism balances exploration and exploitation over time, making it effective for hyperspectral band selection. MFO has shown strong performance on datasets like Indian Pines and Salinas, often outperforming GA and PSO in classification accuracy. However, its local search capability is inherently limited, which can lead to stagnation without hybridization or diversification mechanisms. Additionally, its computational cost increases with population size and data dimensionality, potentially affecting scalability. Despite these limitations, MFO introduces a novel bioinspired search strategy that has broadened the scope of nature-inspired methods in hyperspectral analysis.
• Wild Horse Optimizer (WHO) is a recent metaheuristic inspired by herd dynamics and leadership behaviors in wild horse populations. It simulates population-based search through social interaction and adaptive movement, making it applicable to hyperspectral band selection, where fitness is typically based on classification accuracy.

However, the original WHO faces key limitations, including premature convergence and poor diversity maintenance in later stages. Enhanced variants like IBSWHO ^38^ address these issues using Sobol sequence initialization, Cauchy mutations, and dynamic search strategies to improve exploration and escape local optima. While IBSWHO shows strong performance on standard hyperspectral datasets, these enhancements increase computational cost and parameter tuning complexity—highlighting the trade-offs in designing adaptive metaheuristics for high-dimensional data.

• Binary Multi-objective Clonal Algorithm (BMCA) is inspired by immune system principles, particularly clonal se-lection and affinity maturation. It encodes band subsets as binary strings and simultaneously optimizes objectives such as maximizing entropy and minimizing Pearson correlation. ^39^ Through cloning and hypermutation, BMCA enhances diversity and solution refinement, showing strong classification performance over methods like NSGA-II, BSSO, and PCA in hyperspectral segmentation tasks. However, its limitations include higher computational com-plexity due to mutation and cloning operations, and limited empirical validation compared to more established algorithms. Despite this, BMCA broadens the optimization landscape with a biologically inspired multi-objective framework that addresses trade-offs often overlooked by single-objective approaches.
• Quantum Annealer (QA)-based metaheuristics represent a novel approach to hyperspectral band selection by for-mulating it as a Quadratic Unconstrained Binary Optimization (QUBO) problem, leveraging quantum superposition and tunneling to escape local minima. ^40^ Integrated quantum classifiers like QBoost enable simultaneous band selection and classification within a quantum-enhanced framework. On datasets such as AVIRIS Indian Pines, QA methods have shown competitive or superior performance compared to classical metaheuristics. How-ever, their adoption is hindered by hardware limitations, sensitivity to noise and errors, and limited accessibility and expertise in quantum-hyperspectral integration. Scalability and hybrid quantum–classical approaches remain key areas for future exploration, positioning QA as a promising but currently constrained frontier in hyperspectral optimization.

Based on the related work, we identified the following research gaps: no single metaheuristic algorithm is sufficiently efficient in maintaining a proper balance between exploitation and exploration. Additionally, most metaheuristic algorithms require multiple control parameters, and effective tuning of these parameters remains a significant challenge. Another prevalent issue with swarm-based metaheuristic algorithm is slow or premature convergence. To address these issues, we employed the Jaya optimization algorithm integrated with a mutation operator to enhance the balance between exploration and exploitation. Furthermore, the inclusion of the mutation operator contributes to improving the convergence speed.

## Methodology

### Background

Consider a hyperspectral image dataset represented as $X^{H\times W\times N}$ , where *H* and *W* refer to the spatial dimensions that is height and width of the image, and *N* indicates the number of spectral bands, forming the feature dimension along the third axis. If the spatial pixel position is p(i,j) , then its spectral feature vector is defined as:

$$\left[p_{1}\left(i,j\right),p_{2}\left(i,j\right),p_{3}\left(i,j\right),\ldots \ldots ,p_{N}\left(i,j\right)\right]$$

where each $p_{k}\left(i,j\right)$ corresponds to the intensity value at band *k* for pixel at location ( *i*, *j*), and *k* = 1, 2, …, *N* represents the band index.

Let the hyperspectral data consist of *K* classes, denoted as:

$$\left[C=C_{1},C_{2},C_{3},\ldots ..,C_{K}\right]$$

Let *n* be the number of selected spectral bands, denoted as:

$$\left[B=b_{1},b_{2},b_{3},\ldots ..,b_{K}\right]$$

A hyperspectral image dataset comprising *N* spectral bands and spatial dimensions of *H×W* pixels can be mathematically represented as:

$$\left[C=x_{1},x_{2},x_{3},\ldots ..,x_{N}\right]$$

where each band *xi* can be expressed as a vector of all its pixel values:

$$\left[x_{i}=p_{i}\left(1,1\right),p_{i}\left(1,2\right),\ldots ,p_{i}\left(H,W\right)\right]$$

The following subsection elaborates on the proposed Enhanced Jaya algorithm and its associated objective function.

### Opposition-Based Learning

Opposition-Based Learning (OBL) is a method applied during the initial stage of optimization to generate a diverse and well-distributed set of potential solutions, enhancing the algorithm’s ability to thoroughly explore the search space. Instead of relying solely on random initialization, OBL evaluates each solution alongside its corresponding opposite within the defined search space. Thereby increasing the likelihood of initial population closer to the global optimum. Consider a search space defined for each dimension *j ϵ {*1, 2, …, *d}* by a lower bound $\;a_{j}$ and an upper bound $b_{j}$ . Given a candidate solution $\left[x=x_{1},x_{2},x_{3},\ldots ..,x_{d}\right]$ , the corresponding opposite solution is evaluated as $\left[\bar{x}={\bar{x}}_{1},{\bar{x}}_{2},{\bar{x}}_{3},\ldots ..,{\bar{x}}_{d}\right]$ , where each component ${\bar{x}}_{j}$ is calculated using the formula:

$${\bar{x}}_{j}=a_{j}+b_{j}-x_{j},\text{for}\;j=1,2,\ldots ,d$$ (1)

This equation effectively reflects each component *x j* across the midpoint of the interval $\left[a_{j},b_{j}\right]\;$ thereby generating a solution on the opposite side of the search space.

For each randomly generated solution $x_{i}$ in the initial population, its opposite ${\bar{x}}_{i}\;$ is also computed. The fitness values of both $x_{i}$ and its opposite ${\bar{x}}_{i}\;$ are evaluated, and the one with better fitness is chosen to be included in the population. This method enhances the variety of the starting population and raises the chances of beginning the search near the global optimum.

### Jaya with mutation

The standard Jaya algorithm ^41^ is a population-based metaheuristic that draws inspiration from the concept of “survival of the fittest”. Essentially, the Jaya algorithm seeks to achieve success by getting closer to optimal solutions and avoiding failure by distancing itself from poor ones. It offers several advantages, such as being simple to implement and not requiring any algorithm-specific parameters. Its performance relies only on two factors: the size of the population and the number of iterations. May struggle with local optima in complex, high-dimensional environments like hyperspectral data. To address this limitation, we propose an improved version of the algorithm that incorporates a mutation operator to boost exploration and avoid stagnation.

**Figure 1.**
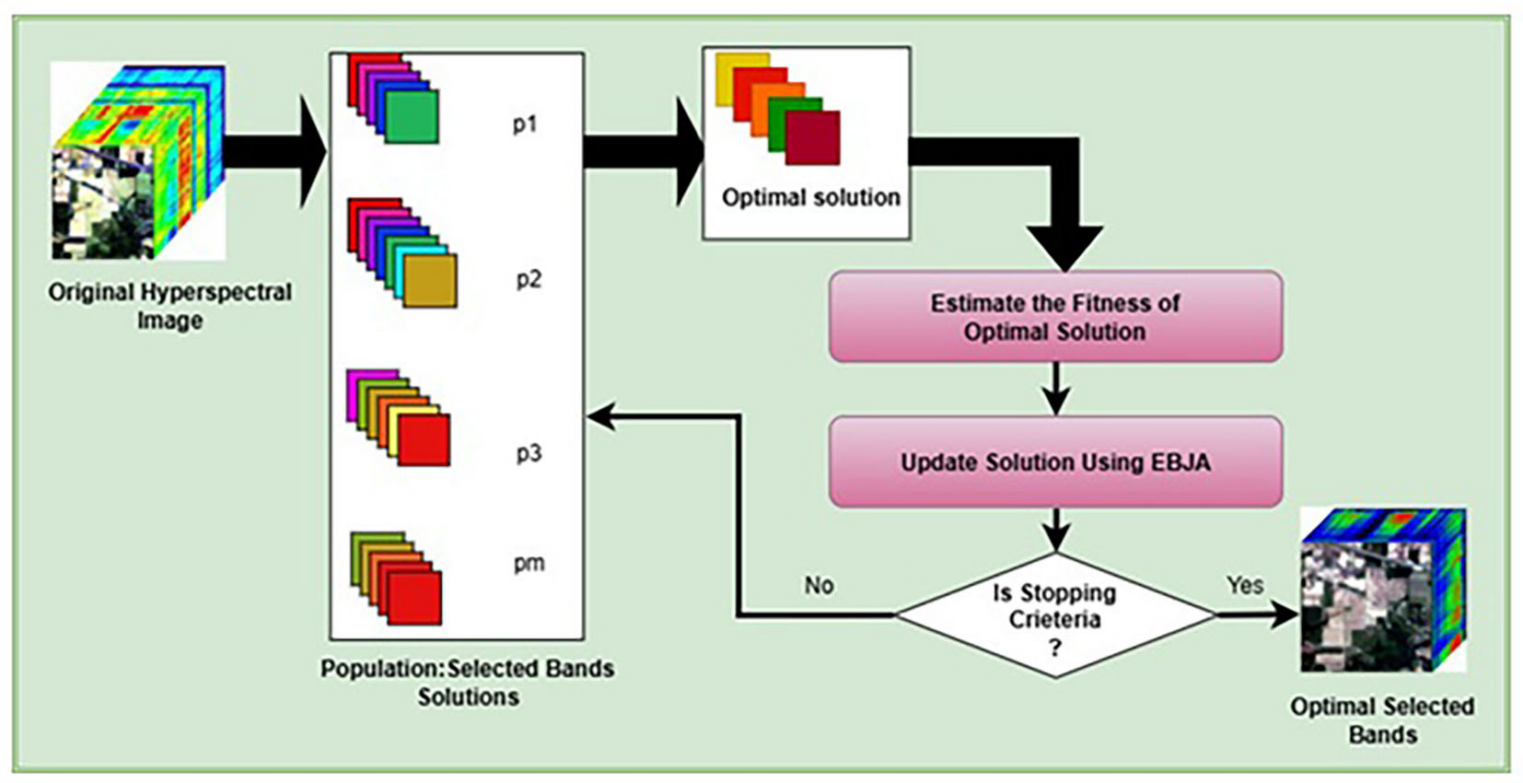
Metaheuristic-based band selection framework.

The key steps of the algorithm represented in the Figure 1 are as follows:

1. Population Initialization: A population of binary vectors is randomly initialized, with each vector representing a potential solution, and the *D*-dimensional vector $\left[x_{i}=X_{\left(i,1\right)},X_{\left(i,2\right)},\ldots ,X_{\left(i,D\right)}\right]$ denoting the $i^{\mathrm{th}}$ solution. These solutions are generated randomly. Each element in the binary vector corresponds to a spectral band, with 1 indicating selection and 0 indicating exclusion.

$$X_{i,j}^{\left(g+1\right)}=X_{i,j}^{\left(g\right)}+r_{1,i,j}\left(X_{\text{best},j}^{\left(g+1\right)}-\left|X_{i,j}^{\left(g\right)}\right|\right)-r_{2,i,j}\left(X_{\text{worst},j}^{\left(g+1\right)}-\left|X_{i,j}^{\left(g\right)}\right|\right)$$ (2)

where,

•
$X_{i,j}^{\left(g\right)}$ represents the value of the $j^{\mathrm{th}}\;$ variable in the $i^{\mathrm{th}}\;$ candidate solution at generation g.
•
$X_{i,j}^{\left(g+1\right)}$ the updated value assigned to the $j^{\mathrm{th}}$ variable of the $i^{\mathrm{th}}\;$ solution in generation *g* + 1.
•
$X_{\text{best},j}^{\left(g+1\right)}$ represents the $j^{\mathrm{th}}$ component of the best candidate solution in generation *g.*
•
$X_{\text{worst},j}^{\left(g+1\right)}$ represents the $j^{\mathrm{th}}\;$ component of the worst candidate solution in generation *g.*
•
$r_{1,i,j},r_{2,i,j}$ are independently generated two random values within the range [0,1] for each index pair ( *i*, *j*).

For each generation, the algorithm identifies the best and worst solutions in the population. New solutions are generated by updating the current population based on the best and worst solutions. In particular, the Jaya algorithm updates each solution by steering it toward the top-performing solution (best candidate) and away from the worst, as described in Eq. (2). To promote faster exploration and convergence toward the optimal solution, an efficient mutation strategy ^42^ is integrated into the Jaya algorithm to mitigate slow convergence. Experimental results demonstrate that integrating a mutation operator into the Jaya algorithm improves the diversity of candidate solutions and enhances the search capability of the algorithm. Consequently, the mutation operator accelerates convergence, allowing the algorithm to reach the optimal solution in fewer iterations.

An adaptive mutation operator is introduced to improve the balance between exploration and exploitation while also reducing the overall computation time. The mutation operator is mathematically defined as follows:

$$X_{i}^{\left(g+1\right)}=X_{r_{1}}^{\left(g\right)}+F.\left(X_{r_{2}}^{\left(g\right)}-X_{r_{3}}^{\left(g\right)}\right)$$ (3)

where

• *j ϵ {*1, 2, …, *D}*, denoting the $j^{\mathrm{th}}\;$ candidate solution in a population of size *D.*
•
$X_{r_{1}}^{\left(g\right)},X_{r_{2}}^{\left(g\right)},X_{r_{3}}^{\left(g\right)}\;$ are randomly selected individuals from the current population at generation *g.*
• *F* is a scaling factor that controls the amplification of the differential variation and avoid stagnation in the population.

An adaptive scaling factor is employed, defined as follows:

$$F=I+\mathrm{rand}.\left(\frac{G-g}{G}\right)$$ (4)

where *I* denote the initial value of the scaling factor *F* which sets to 0.8, The variable *g* refers to the current iteration number, and *G* represents the total number of iterations. The mutation rate gradually declines from 1 to 0 over the course of iterations. It begins with a lower intensity in the initial phases and gradually increases during the later stages. This adaptive adjustment improves the algorithm’s exploration capability. Adaptive scaling allows the mutation intensity to vary dynamically during the optimization process. Larger mutation steps in the early iterations enhance global exploration, while smaller steps in later stages support fine-tuned exploitation and convergence toward the optimal solution. As a result, the algorithm effectively balances exploration and exploitation by incorporating both adaptive mutation and update mechanisms.

### Quasi-reflection reinitialization

This mechanism kicks in when the algorithm seems to stop making progress, usually noticed when there is no noticeable improvement in the objective function after a certain number of continuous iterations. The objective is to introduce complementary or diverse candidate solutions that can guide the search for local optima. Given a solution vector $\left[x=x_{1},x_{2},x_{3},\ldots ..,x_{d}\right]$ , in a bounded search space where $x_{j}\;\epsilon \left[a_{j},b_{j}\right]\;$ , its quasi-reflected counterpart $\left[\hat{x}={\hat{x}}_{1},{\hat{x}}_{2},{\hat{x}}_{3},\ldots ..,{\hat{x}}_{d}\right]$ is computed as:

$${\hat{x}}_{j}=r.\left(a_{j}+b_{j}\right)-x_{j}\;\text{for}\;j=1,2,\ldots ,d$$ (5)

where:

• *r ϵ* [0,1] is a randomly chosen scaling factor that controls the degree of reflection,
•
$a_{j}\;$ and $b_{j}$ are the boundaries (lower and upper) of dimension *j*,
•
$x_{j}$ is the current component of the solution vector.

Unlike strict reflection (which uses a fixed midpoint), quasi-reflection introduces controlled randomness via the factor *r*, thereby generating diverse and non-symmetric alternatives. This strategy is particularly useful for reinitializing poor solutions or revitalizing the population when the algorithm stagnates.

### Objective function

The objective function serves an essential function in evaluating candidate subsets and quantifying their quality. Broadly, it can be divided into two main categories ^43^: filter methods, which evaluate subsets of features based on their intrinsic properties, such as statistical measures without considering the specific classification model, and wrapper methods utilize a classifier to assess the performance of feature subsets, making the evaluation dependent on the chosen model. In our study, the fitness function is designed to integrate two critical objectives: classification accuracy and class separability. This dual-objective approach ensures a balance between the predictive performance of the selected subset and its ability to distinguish between classes effectively. The objective function suggested is typically formulated in the following manner:

$$f\left(b\right)=w_{1}\times \text{Classification Accuracy}+w_{2}\times \text{Measure of Class Separability}$$ (6)

### Metric for classification performance

Accuracy is calculated by dividing the number of correctly classified pixels by the total number of pixels in the test set, offering a clear measure of the model’s overall performance.

$$\text{Accuracy}=\frac{\sum_{x\in \mathrm{TS}}I\left[\hat{y}\left(x\right)-y\left(x\right)\right]}{\left|\mathrm{TS}\right|}$$ (7)

here, I[.] is the indicator function that outputs 1 if a pixel is classified correctly and 0 otherwise. TS represents the set of test pixels. The accuracy function calculates the rate at which pixels are correctly classified. It is essential to note that this rate will be calculated exclusively for the selected band (where $b_{i}=1$ ), while bands with $b_{i}=0$ will be ignored. Here we propose the concept of a KNN classifier to determine the classification accuracy, which reflects the capacity of the selected bands to distinguish between different classes based on their spectral signatures.

### Metrics for class separability

The main objective of class separability within feature selection is to highlight features that most clearly separate classes, determined by evaluating the distance between class distributions. There are various distance metrics, among which the following are applied most frequently in hyperspectral band selection.

The statistical technique Jeffries-Matusita (JM) distance ^44, 45^ is commonly used to assess the degree to which the spectral signatures of different classes are separated in the reduced feature space. It helps determine how effectively two classes (or distributions) can be distinguished based on their statistical characteristics. In the context of binary classification, the JM distance separating class $c_{1}$ from class $c_{2}\;$ is defined as:

$${\mathrm{JM}}_{b_{i}}=\sqrt{2\left(1-e^{-B_{b_{i}}}\right)}$$ (8)

where $B_{b_{i}}$ represents the Bhattacharyya distance between the two classes. The Bhattacharyya distance quantifies the degree of overlap between the two probability distributions and is expressed as:

$$B_{b_{i}}=\frac{1}{8}{\left(\mu_{c_{1}-}\mu_{c_{2}}\right)}^{T}{\left(\frac{\sum_{c_{1}}+\sum_{c_{2}}}{2}\right)}^{-1}\left(\mu_{c_{1}-}\mu_{c_{2}}\right)+\frac{1}{2}\ln\left(\frac{\left|\frac{\sum_{c_{1}}+\sum_{c_{2}}}{2}\right|}{\sqrt{|\sum_{c_{1}}}|+\sqrt{|\sum_{c_{2}}}|}\right)$$ (9)

• μ are the mean vectors of the two distributions (representing the classes).
• *Σ* are the covariance matrices of the distributions.

In the context of multiclass classification, the Jeffries–Matusita (JM) distance is computed using the following formula:

$$D_{b_{i}}=\sum_{i=1}^{N}\sum_{j=1}^{N}k\left(\omega_{i}\right)k\left(\omega_{j}\right){\mathrm{JM}}_{b_{i}}$$ (10)

where k( *ϖ*) with the class prior probability specified, the JM distance for the selected features is defined by:

$$f_{\mathrm{JM}}\left(B\right)=\frac{\sum_{i=1}^{N}b_{i}\times D_{b_{i}}}{\sum_{i=1}^{N}D_{b_{i}}}$$ (11)

The Jeffries-Matusita (JM) distance assumes that the features within each class are distributed according to a Gaussian model.

The Hausdorff distance, ^46^ is a metric used to quantify the similarity between two sets. It quantifies the separation by measuring the distance from the most distant point in one set to the closest point in the other. The Hausdorff distance is utilized to evaluate how well different features (or bands) separate classes.

The Hausdorff distance is used in a binary classification scenario with two classes, $c_{1}$ and $c_{2}$ , is defined as $D_{H}\left(c_{1},c_{2}\right)$ as in below equation:

$$D_{H}\left(c_{1},c_{2}\right)=\max\left\{h\left(c_{1},c_{2}\right),h\left(c_{1},c_{2}\right)\right\}$$ (12)

$$h\left(c_{1},c_{2}\right)=\max_{x_{i}\in c_{1}}\min_{x_{j}\in c_{2}}|x_{i}-x_{j}|$$ (13)

In this context, $x_{i}$ refers to the pixels assigned to class $\;c_{1}$ , whereas *x j* corresponds the pixels assigned to class $c_{2}$ . The function $h\left(c_{1},c_{2}\right)$ refers to the directed Hausdorff distance from *c*_1_ to *c*_2_. But for multiclass problem it can be defined for band *bi* as follows:

$$D_{b_{i}}=\frac{1}{k\left(k-1\right)}\sum_{i=1}^{k-1}\sum_{j=1+1}^{k}D_{H}\left(c_{1,}c_{1}\right)$$ (14)

For the selected features, the HD is calculated as follows:

$$f_{H}\left(B\right)=\frac{\sum_{i=1}^{N}b_{i}\times D_{b_{i}}}{\sum_{i=1}^{N}D_{b_{i}}}$$ (15)

For the selected bands Hausdorff measure is computed by $\sum_{i=1}^{N}b_{i}\times D_{b_{i}}\;$ and for all bands by $\sum_{i=1}^{N}D_{b_{i}}$ . Since $b_{i}$ is binary the band is chosen when $b_{i}=1$ and not selected when $b_{i}=0$ *.*

### Proposed objective function

We put forward three objective functions *f* ( *A*), *f* ( *H*), and *f* ( *JM*) in our model. The classification accuracy is the first objective function.

$$f_{1}\left(b\right)=f_{A}\left(b\right)$$ (16)

The combined value of all objective functions, calculated as a weighted sum, is expressed mathematically as:

$$f\left(X\right)=\omega_{1}f_{1}+\omega_{2}f_{2}+\omega_{3}f_{3}+\ldots +\omega_{m}f_{m}$$ (17)

Subject to $\omega_{1}+\omega_{2}+\omega_{3}+\ldots +\omega_{m}=1$ .

*w*here $\;\omega_{1},\omega_{2},\omega_{3},\ldots ,\omega_{m}$ represent non-negative weights allocated to the *m* objective functions. They can be used to define the second objective by balancing classification accuracy with the Hausdorff term through a weighted sum. Similarly, the third objective function is formed by computing the weighted sum of classification accuracy and the Jeffries–Matusita (JM) distance.

$$f_{2}\left(b\right)=\omega_{A}f_{A}\left(b\right)+\omega_{H}f_{H}\left(b\right)$$ (18)

$$f_{3}\left(b\right)=\omega_{A}f_{A}\left(b\right)+\omega_{\mathrm{JM}}f_{\mathrm{JM}}\left(b\right)$$ (19)

The weight $\omega_{A}$ corresponds to the classification accuracy rate, while $\;\omega_{H}$ and $\omega_{\mathrm{JM}}$ represent the weights for the Hausdorff and JM distances term, respectively. The balance between these functions can be adjusted, or one term can be prioritized over the others.

### Proposed Enhanced Jaya (EJaya)

The proposed Enhanced Jaya algorithm with opposition-based learning, mutation and quasi-reinitialization is described in Algorithm 1 and step-by-step explanation is described in the following three sub-sections.

• **Selection of Population using OBL**
  Initial population is generated by random selection of *M* candidate solutions (band subsets). Each solution $X_{i}$ is represented as a binary vector of length *D*, corresponding to the total number of spectral bands. In this binary encoding, each bit $X_{i,j}\;\epsilon \;\left\{1,0\right\}$ indicates whether the $j^{\mathrm{th}}$ spectral band is selected (1) or not selected (0). By applying Opposition-Based-Learning (OBL) during initialization, the method aims to increase diversity and improve the coverage of potentially optimal regions within the search space right from the beginning. For each randomly generated candidate solution $X_{i},$ its opposite ${\tilde{X}}_{i}$ is also computed using Eq. (1). After evaluating the fitness of both $X_{i},$ and its opposite ${\tilde{X}}_{i}$ , the one with the better result is included in the starting population. This strategy helps to accelerate convergence and avoid premature stagnation by promoting broader coverage of the search space.
• **Evaluation of Fitness**
  It is performed by evaluating each of the candidate solution using a wrapper-based objective function that balances classification accuracy and spectral separability as defined in Eq. (19).
• **Execution of Jaya Optimization with Mutation**
  The main optimization loop proceeds iteratively, the process continues until it either reaches the predefined generation limit *G* or meets the early stopping condition. During each generation, the algorithm identifies the best solution $\;X_{\text{best}}$ , which has the highest fitness, and the worst solution $X_{\text{worst}}$ , which has the lowest fitness, from the current population. Using the Jaya algorithm’s update rule as in Eq. (2), each candidate solution $X_{i}$ is adjusted to move closer to the best solution and further from the worst. For each bit, $r_{1}$ and $r_{2}$ are two random numbers generated from a uniform distribution in [0,1]. To promote exploration, mutation operator is applied with a mutation probability $p_{\mathrm{mut}}<0.2$ as per the Eq. (3). This mechanism helps the algorithm avoid premature convergence and escape local optima.
  The best solution from the previous generation is preserved to ensure that it is not lost due to mutation or other updates. If $\;X_{\text{best}}$ is not present in the current population after updates, it replaces the current worst solution.
• **Mechanism for Diversification**
  To avoid premature convergence, Quasi-Reflection Reinitialization ^47^ method is used. The population may get trapped in a local optimum, resulting in stagnation with no improvement in the best solution over multiple generations. To address this, we introduce such diversification strategies to explore new regions of the solution space that have not yet been examined. If the best fitness has not improved for *P*max consecutive generations, a diversification step is triggered. For each solution $\;X_{i}$ , its opposite solution $X_{\mathrm{opp}}^{i}$ is generated using Eq. (5). If $f\left(X_{\mathrm{opp}}\right)>f(X_{i}$ ), then $X_{i}$ is replaced with its opposite. This approach increases diversity and encourages exploration of unexplored regions of the search space.
• **Termination and Optimal Band Selection**
  After updating and possibly mutating the population, the fitness values of the new candidate solutions are recalculated. The generation counter is then incremented (iter = iter + 1). The algorithm terminates when either the maximum number of generations *G* is reached or no improvement in the best solution is observed for *P*max consecutive generations. Finally, the algorithm returns the best solution $X_{\text{best}}$ discovered during the search process. The optimal set of spectral bands is represented by this binary vector.

Algorithm 1. **Enhanced JAYA algorithm for hyperspectral band selection.**

1: **Initialize** random population ${\left\{P_{i}\right\}}_{i=1}^{\mathrm{Pop}\_\text{size}}\;$ within bounds.

2: **Generate** opposite population ${\bar{P}}_{i}$ using **Eq. (1)** **.**

3: **Evaluate** fitness values $f(P_{i}$ ) and $f({\bar{P}}_{i}$ ) for all *i.*

4: **Merge and Select** best Pop_size individuals from $\left\{P_{i}\right\}\cup \left\{{\bar{P}}_{i}\right\}$ *.*

5: **Set** global best $P^{*}$ from initial population.

6: **for** each generation *t* = 0 to Max_iter *−*1 **do**

**7:   for each individual i = 1 to Pop_size do**

8: **Compute fitness**: fitness ← $f\left(P_{i}\right)$

9 **:     Update solution using** **Eq. (2)**

**10:**  Compute adaptive mutation factor r using **Eq. (4)**

11:  if r<mutation_rate then

12: **Perform mutation** using **Eq. (3)**

13:  if $\text{current best fitness}>f\left(P^{*}\right)$ then

14: **Update** $P^{*}$

15:    noImprove ←0

16:  else

17:    noImprove ←noImprove+1

18:  if noImprove ≥ patience then

19: **Quasi-Reinitialize** population using **Eq. (5)**

20: **Re-evaluate** fitness values

21:   noImprove ←0

22: **Return best band subset** encoded in P∗

The proposed Enhanced Jaya (EJaya) algorithm introduces several key innovations over the standard Jaya framework to effectively address the complexities of hyperspectral band selection. First, it uses opposition-based learning for initialization, second integrates a tailored mutation operator after the conventional Jaya update, introducing controlled randomness that enhances exploration without compromising Jaya’s parameter-free simplicity. This is a novel extension, particularly within the band selection domain, where such mutation-based diversification is rarely incorporated. Third, EJaya employs a stagnation-aware reinitialization mechanism based on quasi-reflection or opposition-based learning, which dynamically diversifies the population by generating opposite or quasi-opposite solutions when improvement stalls. This helps the algorithm escape local optima and improves search robustness in high-dimensional spaces. Fourth, a multi-objective fitness function combining classification accuracy and spectral separability (Jeffries-Matusita distance) is used to guide selection, ensuring the resulting band subsets are both informative and non-redundant. Collectively, these enhancements preserve the lightweight nature of Jaya while significantly improving its exploration-exploitation balance, making EJaya a distinctive and powerful approach for hyperspectral band selection. The computational cost of the proposed EJaya algorithm is primarily influenced by the **population size**, the **number of iterations**, and the **fitness evaluation process**, which involves KNN-based classification and separability measures. The overall time complexity can be approximated as

O(Pop_size×Max_iter×C)

where *C* represents the cost of evaluating a single solution. Although the inclusion of mutation and opposition-based learning slightly increases the per-iteration cost compared to standard Jaya, these enhancements significantly improve convergence speed and reduce the total number of iterations required to reach an optimal solution. As a result, the overall runtime remains efficient and practical for real-world hyperspectral datasets. Furthermore, the dimensionality reduction achieved through band selection reduces the computational burden of the final classification stage.

**Figure 2.**
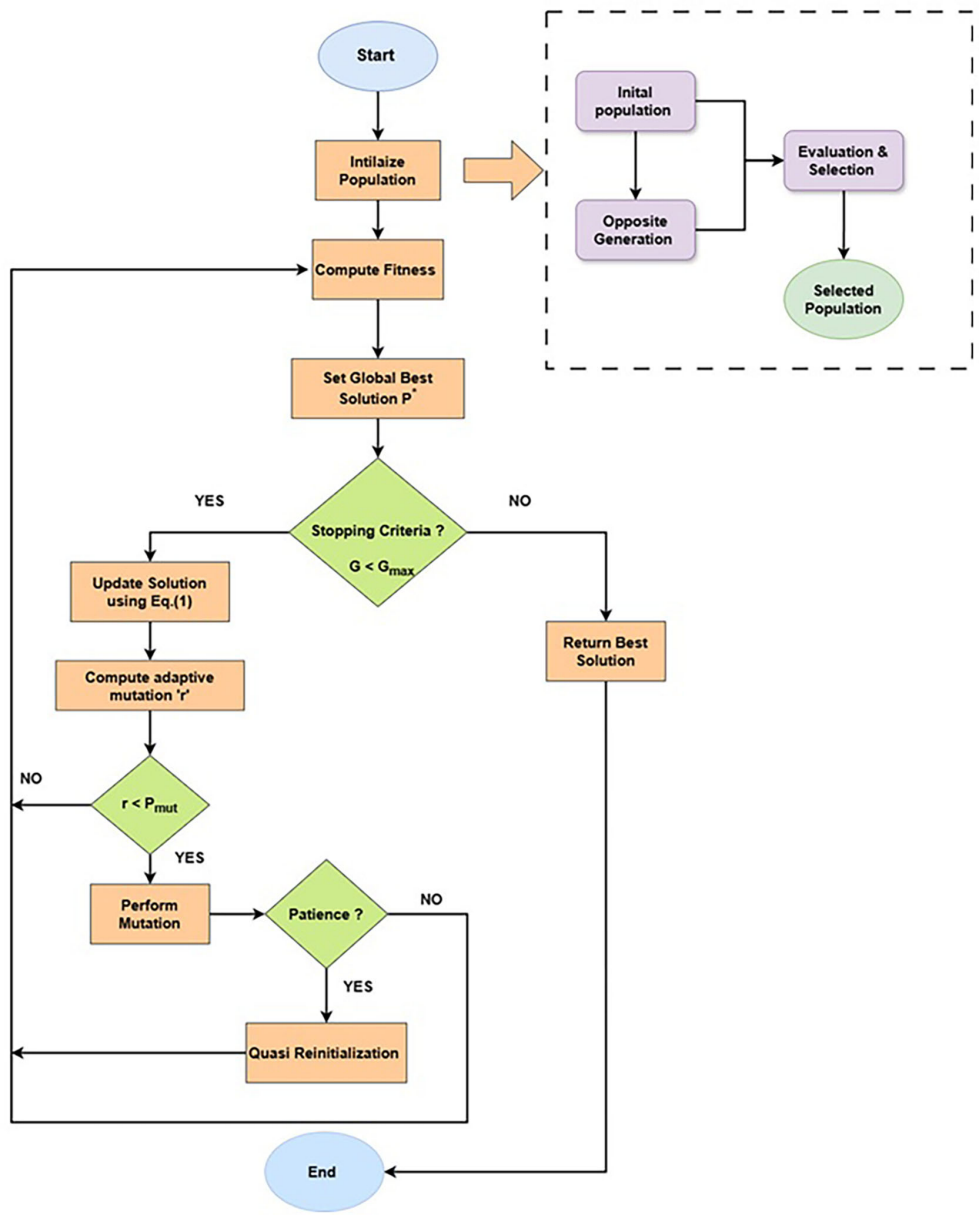
Flowchart of the proposed methodology.

A visual overview of the proposed methodology is shown in the Figure 2.

## Experiment

In this section, Indian Pines (IP), ^48^ Salinas Scene (SA), ^49^ and Pavia University, ^50^ which are three benchmark hyperspectral im-age (HSI) datasets are being used to evaluate the proposed approach. The Table 1 outlines the details of these datasets. The study starts by introducing the datasets, and then provides a detailed analysis of the experiments conducted to identify the most suitable parameters for band selection. A comprehensive comparison was conducted to calculate the efficiency of the proposed algorithm against four other metaheuristic algorithms, emphasizing on overall classification accuracy (OA), average accuracy (AA) and individual class accuracy. Using Python 3.7 the algorithm is implemented, with the simulations performed on a system featuring an Intel Core i7-10870H CPU clocked at 2.21 GHz and 64 GB of RAM. For every dataset, the labeled pixels were split into training and testing sets, with 30% of the samples from each land-cover class randomly selected for training and the remaining 70% used for testing. The HSI data with selected bands were classified using a K-Nearest Neighbors (KNN) classifier. This procedure was carried out 30 times per dataset, and the average results were used for evaluation. In addition, four other metaheuristic-based feature selection techniques: Genetic Algorithm (GA), ^48^ Particle Swarm Optimization (PSO), ^21^ Grey Wolf Optimizer (GWO), ^21^ Modified Grey Wolf Optimization (MGWO) ^31^ and binary JAYA ^41^ were compared with the proposed method and are summarized in Table. ^4– 6^

**Table 1.** Summary of hyperspectral datasets used in the experiments.

Dataset	Spatial dimension	Bands	Classes	Wavelength (nm)	Description	Acquisition
Indian Pines	145 ×145	200	16	400–2500	Agriculture, forest land cover	AVIRIS (Airborne)
Pavia University	610 × 340	103	9	430–860	Urban structures, roads	ROSIS (Airborne)
Salinas	512 × 217	224	16	400–2500	Vegetation, bare soil	AVIRIS (Airborne)

### Dataset

**Indian Pines**: The IP dataset is a significant benchmark in hyperspectral image classification. This is collected by the Airborne Visible Infrared Imaging Spectrometer (AVIRIS) in 1992, the data covers a 145 by 145 square area in Indian Pines, Indiana, USA. The sensor captures wavelengths between 0.4 and 2.5 micrometer. Approximately two-thirds of the area consists of agricultural fields, while the remaining portion features natural landscapes such as forests. The region also includes two major highways, a railway, a few scattered homes, and smaller roads. Since the images were taken in June, early growth of crops like corn and soybeans is visible. After excluding bands related to water absorption, the dataset consists of 200 spectral bands and 16 major land-cover categories, with less than 5% total coverage of the Indianpine dataset.

**Pavia University**: The Pavia University (PU) dataset, another key resource for hyperspectral image classification, was captured in 2002 using the ROSIS sensor. Initially containing 115 spectral bands and covering a 610 by 610-pixel area, the dataset was refined by removing less useful samples, resulting in 103 bands and an area of 610 by 340 pixels. The images have a spatial resolution of 1.3 meters and feature 9 distinct categories.

**Salinas**: The Salinas (SA) dataset was collected using the AVIRIS sensor over the Salinas Valley in California, USA. It consists of 224 spectral bands, covering an area of 512 lines by 217 samples, with a spatial resolution of 3.7 meters. Some bands (108–112, 154–167, and 224) were excluded due to water absorption. The dataset is presented as radiometric data from the sensor, covering categories such as vegetables, bare soil, and vineyards. In Table 2 the first row shows the different datasets, the second row lists the objective functions, which is followed by the Average Accuracy, Overall Accuracy, and the number of bands selected. The three columns present the results corresponding to each objective function. To determine the classification accuracy rate for all methods, the K-nearest neighbors’ technique is employed. It is evident that, for all the datasets evaluated, the model’s average class accuracy and overall accuracy achieved using the objective function $f_{3}$ surpass those obtained with the other objective functions.

**Table 2.** Performance metrics (AA, OA, and number of bands) across different objective functions and datasets.

Dataset	Objective	Accuracy (AA/OA)	# Bands
Indian Pines	*f*1	0.85/86.76	88
	*f*2	0.86/86.79	100
	*f*3	0.86/87.72	52
Pavia U	*f*1	0.91/93.35	48
	*f*2	0.90/92.45	50
	*f*3	0.92/93.64	26
Salinas	*f*1	0.97/92.82	92
	*f*2	0.96/92.75	145
	*f*3	0.97/93.35	60

The outcomes achieved by the objective function (optimization function) $f_{3}$ , which combines accuracy and JM distance, are superior to those of the objective function $f_{2}$ , which uses accuracy and Hausdorff distance term, across all the hyperspectral images.

Table 3 demonstrates a performance comparison between KNN and EJaya across three benchmark hyperspectral datasets: Indian Pine, Pavia University, and Salinas Scene. Key metrics such as Average Class Accuracy, Model Accuracy, and Number of Bands highlight the advantages of the EJaya algorithm for hyperspectral band selection. The EJaya method consistently outperforms KNN by achieving higher accuracies across all datasets while significantly reducing the number of selected bands. This reduction enhances computational efficiency and focuses on the most informative spectral bands, thereby justifying the effectiveness of EJaya for hyperspectral image classification.

**Table 3.** Comparison of classification performance between KNN and EJAYA across hyperspectral datasets.

Dataset	Method	Accuracy (AA/OA (%))	# Bands
Indian Pines	KNN	77.11/77.51	All
	EJAYA	86.00/87.72	52
Pavia U	KNN	87.69/90.05	All
	EJAYA	92.00/93.64	26
Salinas	KNN	95.20/90.50	All
	EJAYA	97.00/93.35	60

Four popular wrapper methods utilizing meta-heuristic algorithms are evaluated against our method for band selection: GA, ^51^ PSO, ^21^ GWO, ^21^ and Jaya. ^41^ On the objective function $f_{3}$ these meta-heuristic algorithms are applied. The experimental results show that PSO, GWO, and Jaya yield similar classification accuracy across all three hyperspectral datasets, while the EJaya algorithm consistently outperforms them.

### Configuration of the proposed method’s parameters

In contrast to the Jaya ^41^ algorithm, which is highly effective at exploitation (refining solutions by steering them towards optimal results), our proposed algorithm enhances performance by incorporating the mutation operator. This addition enables the algorithm to strike a balance between exploration (global search) and exploitation through mutation. For the Enhanced Binary Jaya Algorithm, Table 4 summarizes the main parameters used. The **population size (N = 30)** ensures sufficient diversity while keeping computation manageable, and the **maximum iterations (G = 100)** determines the stopping condition of the algorithm, ensuring that the optimization process runs for enough generations to reach a stable and near-optimal solution. The **mutation factor (0.1)** provides controlled exploration without excessive randomness. For the classification stage **, k = 5 in KNN** was selected to balance accuracy and stability. These values were chosen based on preliminary testing and commonly used settings in related studies. The weight coefficients *ωA*, *ωH*, and *ωJM* in the objective function are assigned a value of 1. The computation of $f_{a}\left(b\right)$ is carried out using a classifier system. For optimal results, the K-nearest neighbors (KNN) algorithm should be applied with K fixed at 5. The main advantage of the KNN algorithm is that it functions without requiring a training model. We recommend using 30% of the data for the training set and 70% for the test set. To avoid overfitting, different training and test sets are used. The training and test sets are generated randomly for each iteration of the algorithm.

**Table 4.** Experimental parameters.

Parameter	Symbol	Value
Population Size	N	30
Maximum Iterations	G	100
Mutation Factor	—	0.1
k in KNN	k	5

### Comparative analysis for Indian Pines dataset

For the Indian Pines dataset Table 5 showcases the classification outcomes achieved through the proposed band selection approach, including overall accuracy, class-wise accuracy, and the average accuracy across all classes. In the same table, the classification outcomes of other competing methods are also presented for comparison. The outcome shows that the proposed method delivers superior performance, achieving the highest classification accuracy and surpassing other optimization techniques.

**Table 5.** Classification results for Indian Pines dataset across different metaheuristic algorithms.

Class	GA ^48^	GWO ^21^	PSO ^21^	MGWO ^31^	JAYA ^41^	EJAYA
Alfalfa	0.00	0.64	0.61	0.32	0.68	0.86
Corn-no till	0.53	0.59	0.61	0.79	0.81	0.84
Corn-min till	0.28	0.52	0.65	0.64	0.75	0.80
Corn	0.15	0.45	0.46	0.78	0.64	0.76
Grass-pasture	0.82	0.81	0.89	0.91	0.94	0.94
Grass-tree	0.94	0.94	0.93	0.97	0.99	1.00
Grass-pasture-mowed	0.17	0.85	0.70	0.76	0.91	0.88
Hay-windrowed	0.96	0.96	0.98	0.99	0.98	1.00
Oat	0.00	0.50	0.50	0.34	0.62	0.83
Soybean-no till	0.60	0.71	0.70	0.75	0.85	0.86
Soybean-min till	0.74	0.70	0.77	0.88	0.88	0.89
Soybean-clean	0.32	0.44	0.59	0.72	0.65	0.71
Wheat	0.96	0.89	0.90	0.97	0.99	0.98
Woods	0.93	0.90	0.91	0.98	0.97	0.97
Bldgs-grass-trees-drives	0.12	0.39	0.48	0.49	0.54	0.53
Stone-steel towers	0.73	0.94	0.92	0.93	0.89	0.89
AA	52.54	70.35	73.23	82.01	82.51	**86.53**
OA	64.86	70.65	74.55	84.08	85.09	**87.72**
NBS (Bands)	145	101	123	52	89	**52**

In this study, overall accuracy (OA) is used as the objective function for evaluating fitness. As indicated in Table 5 the proposed method notably enhances class-wise classification accuracy as compared to other approaches. For example, the classification accuracy for “Alfalfa” increases by 0.86, “Oats” by 0.83, “Corn-no till” by 0.31, and “Corn” by 0.59 percent. However, the proposed method underperforms in certain individual classes, likely due to the fusion of spectral bands, which can obscure important spectral features of specific land-cover classes. Misclassification may also result from the spectral similarity between some land-cover classes, making differentiation more challenging. Despite these challenges, the overall accuracy (OA) of the Enhanced JAYA algorithm showed a marked improvement, increasing by 22.86%. In terms of aggregate performance, EJAYA achieves the highest Average Accuracy (AA) of 86.53% and the highest Overall Accuracy (OA) of 87.72%, both of which exceed the baseline JAYA, MGWO, and other conventional metaheuristics. Importantly, it does so while using only 52 spectral bands — fewer than other algorithms demonstrating both efficiency and effectiveness. These results confirm the advantages of the proposed enhancements (mutation and quasi-reinitialization) in exploring the solution space more effectively and selecting more discriminative band subsets.

### Comparative analysis for Pavia University dataset

For the Pavia University dataset Table 6 displays the classification result achieved from the proposed band selection method, including metrics such as overall accuracy, per-class accuracy, and the mean accuracy across all classes. In the same table the classification outcomes of other competing methods are also presented for comparison. The out-come shows that the proposed method delivers superior performance, achieving the highest classification accuracy and surpassing other optimization techniques. In this study, overall accuracy (OA) is used as the objective function for evaluating fitness. As indicated in Table 6 the proposed method notably enhances class-wise classification accuracy as compared to other approaches. For example, the classification accuracy for “Gravel” increases by 0.37, and “Bare Soil” increases by 0.23 percent. However, the method exhibits lower performance for some individual classes, likely due to the fusion of spectral bands, which can obscure critical spectral features of certain land-cover types. Additionally, the spectral similarity between certain land-cover classes may cause difficulties in differentiation, leading to misclassification. Nevertheless, the overall accuracy (OA) of the Enhanced JAYA algorithm improved significantly, rising by 9.07%. In terms of aggregate metrics, EJAYA achieves the highest Average Accuracy (AA) of 92.10%, the second-highest Overall Accuracy (OA) of 93.64% (only slightly below MGWO at 93.76%), and uses the fewest number of bands (NBS = 26), outperforming JAYA (56), PSO (60), GA (82), and GWO (59).

**Table 6.** Classification results for Pavia University dataset across different metaheuristic algorithms.

Class	GA ^48^	GWO ^21^	PSO ^21^	MGWO ^31^	JAYA ^41^	EJAYA
Asphalt	0.83	0.88	0.89	0.94	0.93	0.93
Meadows	0.97	0.95	0.91	0.98	0.98	0.99
Gravel	0.44	0.68	0.73	0.74	0.78	0.81
Trees	0.80	0.81	0.89	0.95	0.89	0.91
Painted Metal Sheets	0.99	0.98	0.99	0.99	0.99	0.99
Bare Soil	0.58	0.68	0.70	0.88	0.77	0.82
Bitumen	0.75	0.81	0.81	0.83	0.88	0.91
Self-Blocking Bricks	0.84	0.83	0.82	0.91	0.88	0.88
Shadows	0.10	0.99	0.98	1.00	1.00	1.00
AA	70.23	84.52	85.35	91.60	90.12	**92.10**
OA	84.57	87.24	87.38	93.76	92.10	**93.64**
NBS (Bands)	82	59	60	25	56	**26**

### Comparative analysis for Salinas dataset

For the Salinas dataset Table 7 states the classification results achieved with the proposed band selection technique, including overall accuracy, accuracy for each individual class, and the average accuracy across all classes. In the same table the classification outcomes of other competing methods are also presented for comparison. The outcome shows that the proposed method delivers superior performance, achieving the highest classification accuracy and surpassing other optimization techniques. In this study, overall accuracy (OA) is used as the objective function for evaluating fitness. In Table 7 it is indicated that the proposed method notably enhances class-wise classification accuracy as compared to other approaches. For example, the classification accuracy for “Grapes untrained” increased by 0.08, and “Vineyard untrained” rose increased by 0.14 percent. However, the method exhibits lower performance for some individual classes, likely due to the fusion of spectral bands, which can obscure critical spectral features of certain land-cover types. Additionally, the spectral similarity between certain land-cover classes may cause difficulties in differentiation, leading to misclassification. Nevertheless, the overall accuracy (OA) of the Enhanced JAYA algorithm improved significantly, rises by 4.78%. Classification results for the MGWO algorithm on the Salinas dataset were not available in the original publication and could not be reproduced due to the absence of implementation details and parameters. Therefore, MGWO is excluded from this comparison for this dataset.

**Table 7.** Classification results for Salinas dataset across different metaheuristic algorithms.

Class	GA ^48^	GWO ^21^	PSO ^21^	MGWO ^1^	JAYA ^41^	EJAYA
Broccoli green weeds 1	0.98	0.99	0.99	-	1.00	1.00
Broccoli green weeds 2	0.99	0.99	0.99	-	1.00	1.00
Fallow	0.98	0.99	0.99	-	1.00	1.00
Fallow rough plow	0.99	0.99	0.99	-	0.99	0.99
Fallow smooth	0.95	0.97	0.98	-	0.99	0.99
Stubble	0.99	0.99	0.99	-	1.00	1.00
Celery	0.99	0.99	0.99	-	0.99	1.00
Grapes untrained	0.79	0.79	0.81	-	0.86	0.87
Soil vineyard develop	0.99	0.99	0.99	-	1.00	1.00
Corn green weeds	0.92	0.90	0.96	-	0.95	0.97
Lettuce romaine 4wk	0.92	0.98	0.99	-	0.98	1.00
Lettuce romaine 5wk	0.98	0.99	0.99	-	1.00	1.00
Lettuce romaine 6wk	0.97	0.98	0.98	-	0.99	0.99
Lettuce romaine 7wk	0.93	0.97	0.97	-	0.94	0.97
Vineyard untrained	0.57	0.70	0.70	-	0.71	0.72
Vineyard vertical trellis	0.97	0.99	0.99	-	0.99	0.98
AA	93.19	96.23	96.45	-	96.32	97.75
OA	88.57	90.80	91.70	-	92.06	93.35
NBS (Bands)	150	145	120	-	102	60

^1^ MGWO result not available.

### Result analysis

**Figure 3.**
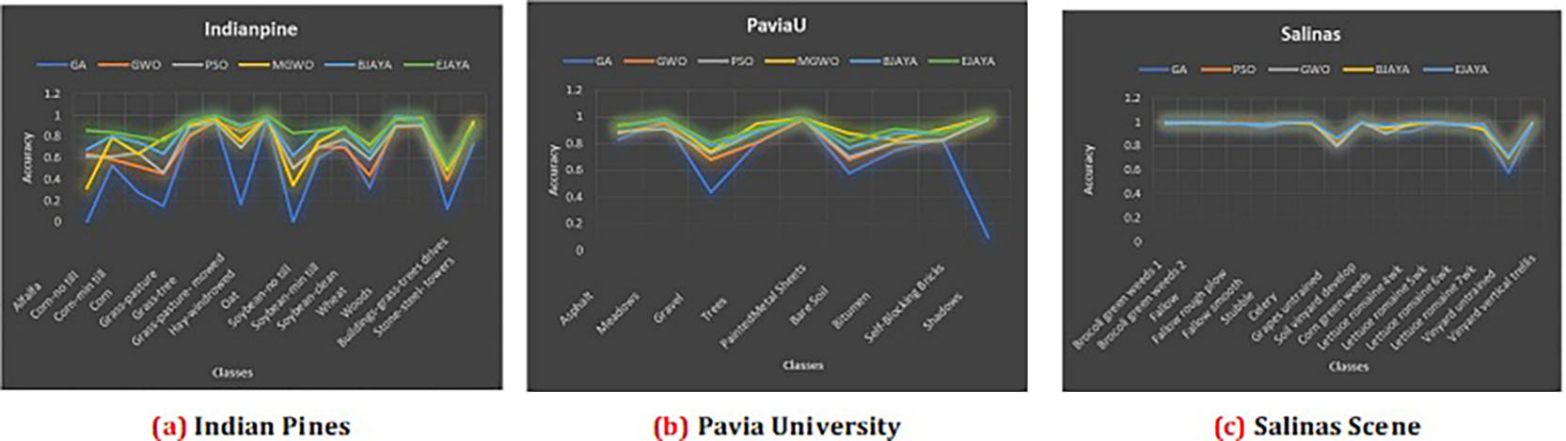
Classification accuracy comparison across different classes for Indian Pines, Pavia University, and Salinas scene datasets.

The resulting graphs shown in Figure 3 illustrate the classification accuracy obtained using five metaheuristic algorithms: Genetic Algorithm (GA), Particle Swarm Optimization (PSO), Grey Wolf Optimizer (GWO), Modified Grey Wolf (MGWO), Binary JAYA (BJAYA), and Enhanced JAYA (EJAYA)—across three hyperspectral datasets. Land cover classes, together with Average Accuracy (AA) and Overall Accuracy (OA), are shown on the x-axes, while the y-axes present the classification accuracy. Performance of EJAYA in all three datasets, consistently outperforms the other algorithms, demonstrating its superior ability to optimize hyperspectral band selection for classification. By achieving the highest scores in both Average Accuracy (AA) and Overall Accuracy (OA), it is recognized as the most reliable method for classifying hyperspectral data. For Indian Pines Dataset EJAYA outperforms others in challenging classes like “Alfalfa” and “Oats,” with substantial improvements in accuracy compared to other algorithms. For Pavia University dataset EJAYA achieves the highest accuracy for difficult classes such as “Shadows” and maintains robust performance across all other classes. For Salinas dataset EJAYA excels in tough classes like “Grapes Untrained” and “Soil Vineyard Develop”, while also providing the best overall classification accuracy.

**Figure 4.**
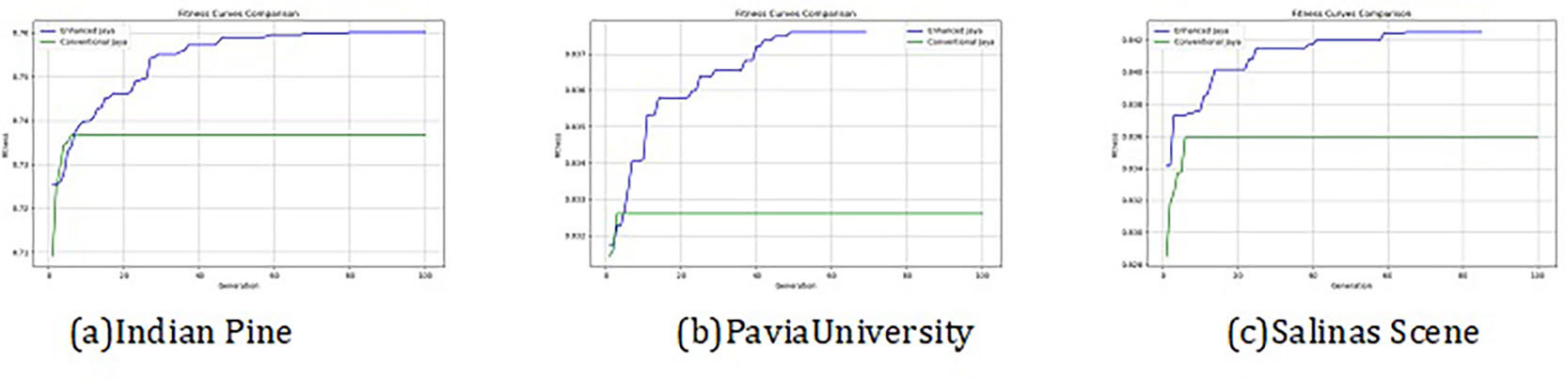
Best fitness curve comparison for Indian Pines, Salinas Scene, and Pavia University datasets.

**Figure 5.**
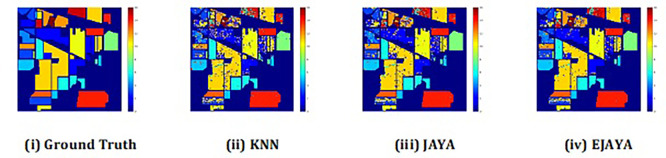
Classification map of Indian Pines dataset using KNN, Jaya, and EJaya.

**Figure 6.**
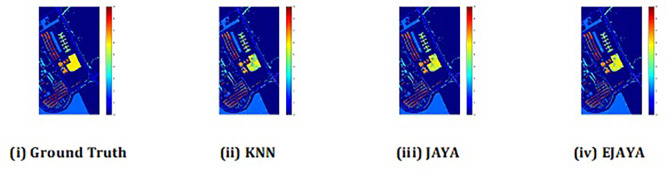
Classification map of Pavia University dataset using KNN, Jaya, and EJaya.

**Figure 7.**
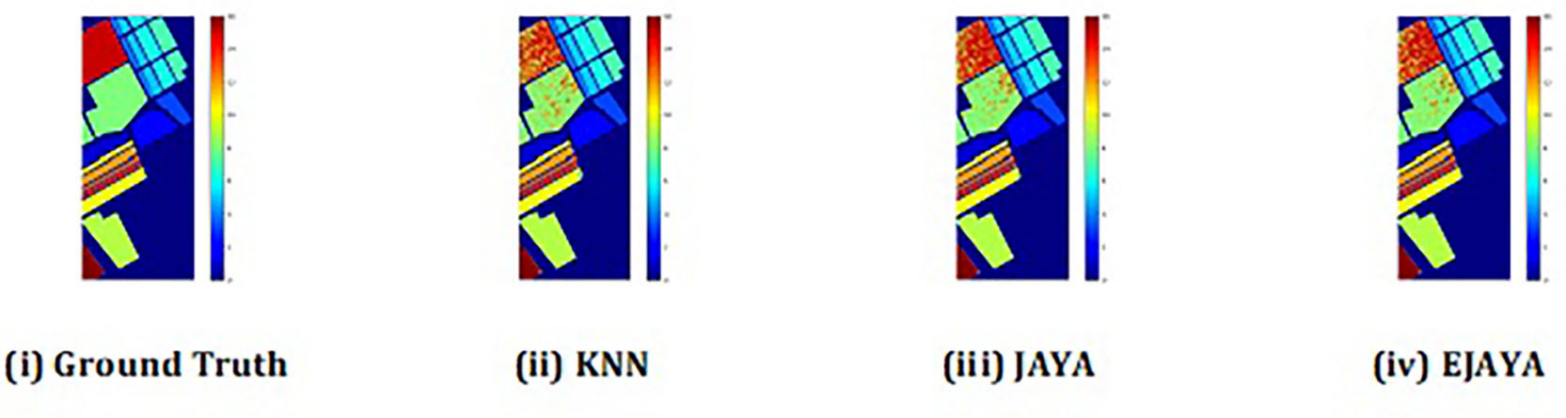
Classification map of Salinas Scene dataset using KNN, Jaya, and EJaya.

The convergence curves shown in Figure 4 indicate that our approach successfully achieved the global optimum across all three datasets. By visual analysis the ground truth map serves as a reference, representing the actual class distribution of the hyperspectral data. It is used to evaluate the accuracy of the algorithms. The classification map generated by JAYA shows a close approximation to the ground truth, but it exhibits some misclassifications in specific areas, especially in regions with overlapping spectral signatures. The EJAYA classification map demonstrates a more refined and accurate representation compared to JAYA, with fewer misclassified pixels. It captures the class boundaries more precisely, particularly in challenging areas. The classification maps for the Indian Pines, Pavia, and Salinas datasets—produced using both the JAYA and EJAYA methods—are shown in Figures 5, 6, and 7.

**Figure 8.**
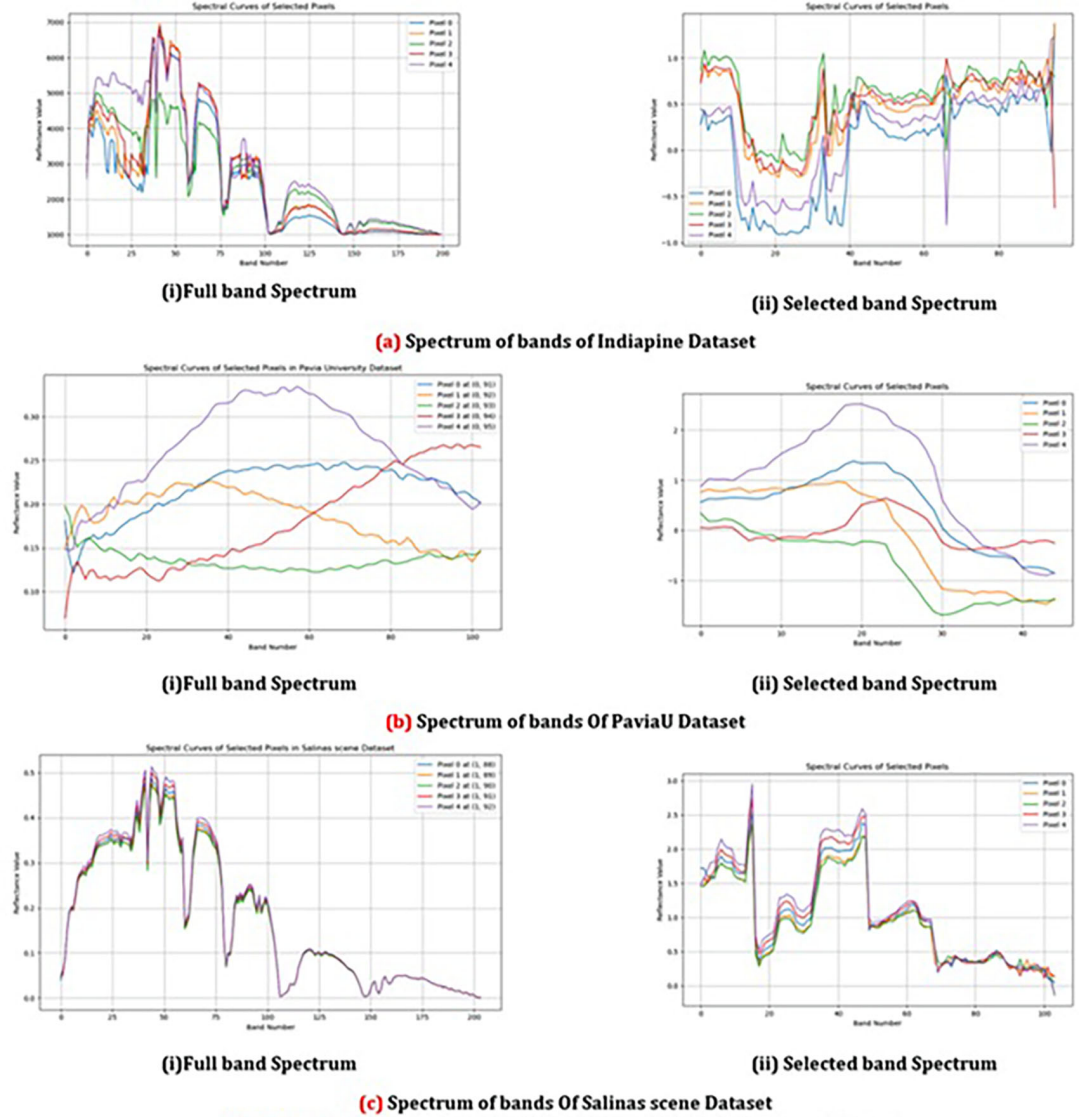
Comparison between spectrum of bands of different dataset.

In Figure 8 it highlights the variability in reflectance values across the selected bands for different pixels in different datasets. It showcases how distinct classes exhibit unique spectral patterns, which is particularly valuable for under-standing class-specific characteristics in hyperspectral data. This visualization aids in highlighting the most relevant bands that contribute to effectively separating different classes. By observing these patterns, effectiveness of band selection techniques and optimize classification performance can be evaluated.

### Ablation study

To thoroughly evaluate the individual contributions of the components integrated into the proposed Enhanced JAYA (Ejaya) framework, an ablation study was carried out by selectively disabling two key strategies:

1. **Opposition-Based Learning (OBL)** mechanism, which aims to produce a uniformly distributed initial population.
2. **Quasi-reflection Reinitialization Strategy**, which is triggered when convergence stagnates or poor solutions persist, thereby supporting a balanced exploration–exploitation process.

To facilitate this analysis, two simplified variants of Ejaya were constructed:

• **Ejaya-NOBL:** This variant excludes the OBL mechanism in the initialization phase.
• **Ejaya-NQRI:** This version removes the quasi-reflection reinitialization component.

The comparative outcomes of these variants along with the complete Ejaya method are summarized in Table 8. To evaluate the effectiveness of the complete Ejaya method, Overall Accuracy (OA) and Number of Bands (NB) are employed as evaluation criteria.

**Table 8.** Ablation study results on Indian Pines, PaviaU, and Salinas datasets. Values are reported as mean ± standard deviation.

Method	Metric	Indian Pines	PaviaU	Salinas
EJaya	OA (%)	**87.28 ± 0.21**	**93.24 ± 0.15**	**92.92 ± 0.10**
	NB	**51.89 ± 2.18**	**25.78 ± 1.84**	**59.35 ± 1.72**
EJaya-NOBL	OA (%)	86.44 ± 0.25	93.61 ± 0.19	94.41 ± 0.11
	NB	70.12 ± 2.21	38.91 ± 2.01	75.51 ± 2.00
EJaya-NQRI	OA (%)	86.37 ± 0.26	93.50 ± 0.21	94.10 ± 0.15
	NB	78.22 ± 2.31	45.12 ± 2.08	88.32 ± 1.78
Jaya	OA (%)	84.92 ± 0.27	91.32 ± 0.30	91.78 ± 0.31
	NB	88.41 ± 2.42	55.21 ± 2.19	101.91 ± 2.21

The ablation results clearly indicate that the complete EJaya method achieves superior fitness values compared to its reduced counterparts. The performance decline in the OBL-removed version (NOBL) can be attributed to reduced population diversity, which increases the risk of premature convergence. Similarly, QRI-removed version (NQRI) strategy diminishes the algorithm’s ability to recover from stagnation, thereby weakening its global search capability. Overall, these findings underscore the critical importance of both the OBL-based initialization and the Quasi-Reflection Reinitialization techniques, along with the mutation-based enhancements in the Jaya algorithm, in improving the effectiveness of band selection and enhancing classification accuracy.

## Conclusion

In this paper, we developed a band selection strategy designed to improve the time efficiency of hyperspectral image classification. The proposed method uses a parameter-free Jaya optimizer with a Differential Evolution mutation operator for band selection. It is further enhanced by opposition-based learning (OBL) initialization and Quasi-Reflection reinitialization to improve exploration and diversity. The objective of incorporation of mutation operator aims to balance the exploitation and exploration within the Jaya algorithm. This improvement of exploration in the Jaya algorithm leads to an enhancement of the optimal selection of minimal bands in the hyperspectral images. Furthermore, OBL and Quasi-Reflection reinitialization strategies employed to improve diversity of solutions in the population that enhance ac-curacy performance and band selection. This study evaluates three objective functions: classification accuracy and two measures of class separability—the Hausdorff distance and the Jeffries–Matusita distance. To showcase the effectiveness of our approach with three objective functions, we conducted experiments using three commonly used hyperspectral image datasets: Indian Pines, Pavia University, and Salinas Scenes. Performance of algorithms are measured using three criteria such as overall accuracy, average accuracy, and individual accuracy in KNN classifier. Our experimental results demonstrate that the objective function based on Accuracy and Jeffries-Matusita distance yields superior classification accuracy across the datasets.

The effectiveness of the proposed method was also evaluated by comparing it with four contemporary techniques from the literature: Genetic Algorithm (GA), Particle Swarm Optimization (PSO), Grey Wolf Optimizer (GWO), and the standard Jaya algorithm. The reported results reveal that the effectiveness of the proposed approach against mentioned methods in terms of the KNN classification accuracy. For future work, we would study different hybridized metaheuristic algorithms to select a minimum subset of bands while preserving classifier performance in hyperspectral image analysis. Further, a novel CNN model could be designed to test our model for optimal band selection.

### Ethics and consent

Ethical approval and consent were not required.

## Data Availability

Datasets used in our experiments are publicly available benchmark hyperspectral datasets. Indian Pines and Salinas datasets are available from the AVIRIS (Airborne Visible/Infrared Imaging Spectrometer) website, NASA Jet Propulsion Laboratory (JPL). Pavia University dataset is available from the Hyperspectral Remote Sensing Scenes (University of Pavia). As these datasets are freely accessible to the research community, we did not generate or own the raw data ourselves. Instead, we used the publicly available versions provided by the respective institutions, and our results (figures and tables) were derived from these datasets. The hyperspectral image datasets used in this study are publicly available and can be accessed as follows:
•
Indian Pines dataset is available from the Purdue University website at http://www.ehu.eus/ccwintco/index.php/Hyperspectral_Remote_Sensing_Scenes#Indian_Pines. ^48^
• Salinas dataset is publicly available at the AVIRIS sensor data source http://www.ehu.eus/ccwintco/index. php/Hyperspectral_Remote_Sensing_Scenes#Salinas. ^49^
• Pavia University dataset can be obtained from the University of Pavia repository at http://www.ehu.eus/ccwintco/index.php/Hyperspectral_Remote_Sensing_Scenes#Pavia_University. ^50^
