# Supplementary material for: An Enhanced Jaya Algorithm with Mutation and Diversity-Preserving Strategies for Hyperspectral Band Selection
Source: F1000Res. 2026 Jan 24;14:999. Originally published 2025 Sep 29. [Version 2] doi: 10.12688/f1000research.167794.2 (PMC12583912; doi:10.12688/f1000research.167794.2)
Supplement: Peer review history

# Reviewer report: Reviewer response for version 1

**DOI:** 10.5256/f1000research.184937.r419016

**Published:** 2025-11-03

**Authors:** Soubhagya Sankar Barpanda

**Affiliations:** 1 VIT-AP University, Vijayawada, Andhra Pradesh, India

The manuscript entitled “Enhanced Jaya Algorithm with Mutation and Diversity-Preserving Strategies for Hyperspectral Band Selection” presents a well-written and technically sound study. The proposed EJaya algorithm demonstrates clear improvements over existing metaheuristics and is evaluated on three widely used hyperspectral datasets. The paper is relevant and of interest to the remote sensing and machine learning community. Overall, the manuscript is suitable for indexing after minor revisions. My detailed comments are as follows:

**1. Clarity & Presentation**

- Improve figure captions, especially for Figures 2–7. Captions should be more descriptive (e.g., “Classification map of Indian Pines dataset using KNN, Jaya, and EJaya” instead of just “Classification Map”).

**2. Methodology**

- It would be helpful to provide a summary table of all experimental parameters (population size, iterations, mutation factor, K for KNN, etc.) in one place for easy reference.
- A short note on the computational cost or runtime complexity of EJaya compared with GA/PSO/Jaya would add clarity. Even approximate runtime per dataset would be valuable.

**3. References & Language**

- Please check for minor typos (e.g., “Opposition-Based Leaning” should be “Learning”).
- Ensure consistent use of acronyms (EJaya, OBL, QRI, OA/AA, etc.) throughout the text.
- It would strengthen the Related Work section to include one or two more recent references (2023–2024) on metaheuristics for hyperspectral band selection.

**Recommendation: Minor Revision**

 The manuscript is strong in terms of methodology and experimental results. Addressing the above points will significantly improve clarity, reproducibility, and readability, making the contribution more accessible to the broader community.

Is the work clearly and accurately presented and does it cite the current literature?

Yes

If applicable, is the statistical analysis and its interpretation appropriate?

Yes

Are all the source data underlying the results available to ensure full reproducibility?

Yes

Is the study design appropriate and is the work technically sound?

Yes

Are the conclusions drawn adequately supported by the results?

Yes

Are sufficient details of methods and analysis provided to allow replication by others?

Yes

Reviewer Expertise:

Image processing, ML/DL applications on image analysis.

I confirm that I have read this submission and believe that I have an appropriate level of expertise to confirm that it is of an acceptable scientific standard.

# Response

**Published:** 2025-12-09

**Authors:** Partha Pratim Sarangi

**Affiliations:** School of Computer Engineering, Kalinga Institute of Industrial Technology, Bhubaneswar, Odisha, India

**Comment 1: Clarity & Presentation**

**Reviewer’s comment:**

 Improve figure captions, especially for Figures 2–7. Captions should be more descriptive (e.g., “Classification map of Indian Pines dataset using KNN, Jaya, and EJaya” instead of just “Classification Map”).

**Response:**

 Thank you for the suggestion. We have revised all figure captions (Figures 2–7) to be more informative and descriptive. Each caption now clearly explains the dataset, method used, and what the figure represents.

**Comment 2: Methodology**

**Reviewer’s comment:**

 Provide a summary table of all experimental parameters (population size, iterations, mutation factor, K for KNN, etc.) in one place for easy reference.

**Response:**

 We agree with the reviewer. A consolidated experimental parameters table has now been added (Table 4 in the revised manuscript). It summarizes algorithmic settings such as population size, number of iterations, mutation factor (DE), K value for KNN.

**Reviewer’s comment:**

 Add a short note on the computational cost or runtime complexity of EJaya compared with GA/PSO/Jaya. Approximate runtime per dataset would be valuable.

**Response:**

 We have added a paragraph explaining about “Computational Cost and Runtime Analysis” in the Methodology section.

**Comment 3: References & Language**

**Reviewer’s comment:**

 Check minor typos (“Opposition-Based Leaning” → “Learning”). Ensure consistent use of acronyms (EJaya, OBL, QRI, OA/AA, etc.).

**Response:**

 We have thoroughly proofread the manuscript and corrected all typographical errors, including the issue mentioned. Acronyms have been standardized throughout the text and verified for consistent usage across sections.

**Reviewer’s comment:**

 Strengthen the Related Work section with one or two recent (2023–2024) references on metaheuristics for hyperspectral band selection.

**Response:**

 We appreciate this helpful suggestion. Already we have added five recent papers from research works (2023–2024) on metaheuristic-based band selection to the Related Work section.

# Reviewer report: Reviewer response for version 1

**DOI:** 10.5256/f1000research.184937.r419019

**Published:** 2025-10-27

**Authors:** Prabhat Kumar Sahu

**Affiliations:** 1 Siksha O Anusandhan (Deemed to be University), Odisha, Bhubaneswar, India

The manuscript presents an Enhanced Jaya (EJaya) algorithm with mutation, opposition-based learning (OBL), and quasi-reflection reinitialization (QRI) for hyperspectral band selection. The study is well-motivated, clearly structured, and demonstrates competitive results across three benchmark hyperspectral datasets (Indian Pines, Pavia University, and Salinas). The proposed improvements address common limitations of standard Jaya and related metaheuristics. The work is promising and deserves indexing after addressing the following minor revisions:

**1. Introduction & Related Work**

- Some parts of the Related Work section are very detailed. Consider adding a summary table of algorithms vs. limitations for quick comparison.

**2. Methodology**

- In the description of the mutation operator (Eq. 3), provide a brief intuition of why adaptive scaling helps balance exploration and exploitation.
- Clarify the stopping criterion: Is termination based on maximum iterations and/or stagnation limit (Pmax)? Please state this explicitly.

**3. Language & Formatting**

- References are generally strong, but please consider citing one or two recent 2023–2024 studies on hybrid Jaya or swarm-based HSI band selection to make the Related Work fully up to date.

Is the work clearly and accurately presented and does it cite the current literature?

Yes

If applicable, is the statistical analysis and its interpretation appropriate?

Yes

Are all the source data underlying the results available to ensure full reproducibility?

Yes

Is the study design appropriate and is the work technically sound?

Yes

Are the conclusions drawn adequately supported by the results?

Yes

Are sufficient details of methods and analysis provided to allow replication by others?

Yes

Reviewer Expertise:

Machine Learning

I confirm that I have read this submission and believe that I have an appropriate level of expertise to confirm that it is of an acceptable scientific standard.

# Response

**Published:** 2025-12-09

**Authors:** Partha Pratim Sarangi

**Affiliations:** School of Computer Engineering, Kalinga Institute of Industrial Technology, Bhubaneswar, Odisha, India

**Comment 1: Introduction & Related Work**

**Reviewer’s comment:**

 Some parts of the Related Work section are very detailed. Consider adding a summary table of algorithms vs. limitations for quick comparison.

**Response:**

 Thank you for the suggestion. Instead of adding a summary table, we have revised the end of the Related Work section to include a brief synthesized paragraph that highlights the key limitations of existing algorithms and how our work addresses them. This addition provides a concise comparative overview while keeping the section streamlined without expanding it further with a table.

**Comment 2: Methodology**

**Reviewer’s comment:**

 In the description of the mutation operator (Eq. 3), provide a brief intuition of why adaptive scaling helps balance exploration and exploitation.

**Response:**

 We appreciate the reviewer’s recommendation. A short explanatory note has been added immediately after Eq. (3), clarifying that adaptive scaling increases mutation intensity during the early search phase to promote exploration, and gradually stabilizes in later stages to encourage exploitation, thereby improving convergence stability.

**Reviewer’s comment:**

 Clarify the stopping criterion: Is termination based on maximum iterations and/or stagnation limit (Pmax)? Please state this explicitly.

**Response:**

 This has now been clearly stated in the Methodology section. The algorithm terminates either when the maximum number of iterations is reached or when the stagnation limit (Pmax) is triggered, whichever occurs first.

**Comment 3: Language & Formatting**

**Reviewer’s comment:**

 References are generally strong, but please consider citing one or two recent 2023–2024 studies on hybrid Jaya or swarm-based HSI band selection to make the Related Work fully up to date.

**Response:**

 We appreciate this helpful suggestion. Already we have added five recent papers from research works (2023–2024) on swarm-based band selection to the Related Work section.
